# Bacterial strains colonizing the sensor electrodes of a continuous glucose monitoring system in children with diabetes

**DOI:** 10.1007/s00592-020-01601-w

**Published:** 2020-09-17

**Authors:** Sebastian Seget, Ewa Rusak, Mirosław Partyka, Ewa Samulska, Aleksandra Pyziak-Skupień, Halla Kamińska, Eliza Skała-Zamorowska, Przemysława Jarosz-Chobot

**Affiliations:** 1 Department of Children’s Diabetology, Medical University of Silesia, Katowice, Poland; 2 Laboratory Diagnostics Centre, Upper Silesian Child Health Centre, Katowice, Poland

**Keywords:** Diabetes type 1, Skin complications, Skin infections, CGM, Continuous glucose monitoring system, Sensor colonization

## Abstract

**Introduction:**

The higher frequency of infections in diabetic patients is caused by a hyperglycemic environment, which promotes immune dysfunction. People with diabetes are more prone to skin infections.

A continuous glucose monitoring (CGM) system provides information on changes in blood glucose (BG) levels throughout the day. Its use facilitates optimal therapeutic decisions for a diabetic patient. One of the factors limiting the use of CGM is inflammation at the insertion site.

**Aim of the study:**

The aim of the study was the microbiological identification of the bacterial strains which are found on CGM sensor electrodes.

**Material and methods:**

We performed microbiological tests on patients′ CGM Enlite Medtronic electrodes, which were removed after 6 days of usage according to the manufacturer′s instructions. 31 sensors were examined from 31 children (14 girls) aged from 0.5 to 14.6 years. The microbiological analysis was routinely performed at the Department of Children’s Diabetology Medical University of Silesia in Katowice, Poland.

**Results:**

12 (39%) of the electrodes were colonized. In 11 (92%) cases the electrodes were colonized by one bacteria strain. 7 times methicillin-sensitive coagulase negative staphylococcus (MSCNS) was detected. We also found one case of *Klebsiella pneumoniae, Ochrobactrum tritici, Bacillus sonorensis* and methicillin-resistant coagulase-negative Staphylococci (MRCNS) colonization. One electrode was colonized by the mixed flora *Enterococcus faecalis*, methicillin-susceptible coagulase-negative Staphylococci (MSCNS), *Pseudomonas stutzeri*, methicillin-susceptible *Staphylococcus aureus* (MSSA). The median HbA1c in the group with colonization of electrodes was 6, 85% (6, 3–7, 6%) versus 6, 3% (5, 8–7, 5%) in the group without colonization. The median BMI in the group with colonization of the electrodes was 17.10 kg/m^2^ (16.28–18.62 kg/m^2^) versus 15.98 kg/m^2^ (15.14–17.96 kg/m^2^) in the group without colonization. Statistically, significantly more frequently electrodes are colonized in older children (median age in the group with colonization of electrodes 11.43 years (6.52–12.27 years), without colonization 8.42 years. (3.098–9.375 years); (*p* = 0.033).

**Conclusions:**

It seems that older children are more likely to have their sensor electrode colonized by bacterial strains.

## Introduction

Diabetes is a group of metabolic diseases. They are characterized by chronic hyperglycaemia as the effect of insufficient insulin secretion, its disturbed action or both.

According to the International Diabetes Federation, the number of children with type 1 diabetes, between 0 and 14 years of age, in the world in 2013 was 497,100 [1]. Chronic complications of diabetes affect many organs. They can be divided into vascular and extravascular complications. Vascular complications include microvascular (retinopathy, neuropathy and nephropathy) and macrovascular (coronary disease, peripheral vascular disease and cerebrovascular disease). It is worth noting that the treatment of diabetes complications costs more than managing the disease itself [2].

Furthermore, extravascular complications include problems such as: weak stomach, sexual dysfunction and skin lesions. About 30% of all diabetic patients will develop skin changes throughout their lives [3].

The high incidence of dermatological disorders among diabetic patients described in the literature confirms the clinical significance and the high impact of this complication. The most commonly reported disorder is infection [4]. Skin infections in diabetic patients are often caused by Staphylococcus aureus [3].

Diabetes is associated with an increased risk of infectious diseases and their complications, and on average double the mortality risk compared to non-diabetic patients. In the course of diabetes, dysfunction of the immune system occurs. This dysfunction includes the reduction of phagocytic activity of neutrophils and macrophages, impairment of NK cell activity and dysfunction of dendritic cells [5].

Continuous glucose monitoring (CGM) provides information on changes in blood glucose throughout the day and facilitates optimal therapeutic decisions for patients with diabetes. Information on trends helps to identify and prevent unwanted periods of hypo- and hyperglycaemia. The use of CGM permits the improvement of blood glucose control [5, 6].

CGM is also an educational tool that reveals the effect of insulin dosing, exercise, food intake and other everyday events such as work, illness and stress on glycemic control [7]. However, it is known, that the accuracy of CGM is insufficient for making treatment decisions, which is the limitation of this type of technology [8].

The CGM consists of three main components: glucose-sensing electrodes measuring the glucose concentration continuously in the interstitial fluid, transmitter that transfer the data to a receiver- display device, that shows blood glucose levels [9].

The principle of the glucose measurements is based on the oxidation of glucose. The glucose oxidase is placed on the electrode surface and catalyzes the chemical reaction of glucose with oxygen to produce an electrical signal. The glucose from interstitial fluid is continuously converted into an electrical signal, which is proportional to the glucose concentration.[10].

## Aim of the study

The aim of the study was to identify the bacterial strains that colonize the sensor electrodes of a CGM system in children with diabetes.

## Material and methods

We performed the microbiological tests of the CGM Enlite Medtronic electrodes in randomly selected patients of the Department of Children′s Diabetology Medical University of Silesia after their expiration period. Sensors were put on for 6 days according to the manufacturer′s instructions. Before applying the CGM sensors, both patients and their parents received training. The sensors were placed on unchanged skin, and no inflammation was observed at the site of the electrode after sensor removal.

Qualified nurses collected material for microbiological tests. The semi-quantitative Maki breeding technique was used to assess microbial contamination of the sensors. The cultures were prepared according to the methods commonly used in microbiology. Bruker MALDI Biotyper was used to identify the strains. Glycemic hemoglobin was measured by HPLC (according to the standards of DDCT).

31 sensors from 31 children (14 girls and 17 boys) with type 1 diabetes, without vascular complications, were examined during the year. The mean age in the study group was 7.93 years (0.5–14.6 years). The median HbA1c in the study group was 7.2% (4.9–13.8%). The mean duration of diabetes was 2.58 years (0.4–9.0 years).

### Statistical analysis

The statistical analysis of the results of the tests obtained was done with STATISTICA 13.1 computer program (Statsoft, Tulsa, OK, USA). Verification of the normality of the distribution was carried out with the use of the W Shapiro–Wilk test. When comparing differences in the range of evaluated parameters between the studied groups, in the case of normal distribution of numerical data the student′s t-test was used. For the non-normal distribution we used the non-parametric Mann–Whitney test.

The chi^2^ test with the Yates correction was used to evaluate the statistical differences in the frequency of infections depending on the sex. The value of *p* < 0.05 was considered to be the statistical significance threshold.

## Results

In 12 patients (39%), colonization of CGM electrodes was observed. The characteristics of the patients in whom we managed to grow bacteria are presented in Table 1.

**Table 1:** The characteristics of the patients with colonization of CGM electrodes

Patient	Sex	Age (years)	HbA1c (%)	BMI (kg/m^2^)	BMI Z-score	Duration of diabetes (years)	Daily dose of insulin (units)	Electrode flora
1	F	3,6	6,8	16,5	0,842	6,6	23,05	MSCNS
2	M	11,2	6,5	19,3	0,776	0,8	18,9	MSCNS
3	F	13,7	6,9	18,9	−0,162	6,0	63,4	MSCNS
4	M	14,7	12,6	16,0	−1,798	1,8	68,45	MSCNS
5	F	10,5	7	14,5	−1,386	4,4	20,2	*Enterococcus faecalis*, MSCNS, *Pseudomonas stutzeri*, MSSA
6	F	11,8	11,8	18,3	0,225	7,0	35,45	*Klebsiella pneumoniae*
7	M	9,5	6,6	16,0	−0,146	1,1	13,425	*Ochrobactrum tritici*
8	M	11,8	6,9	17,0	−0,375	0,4	17,7	MSCNS
9	M	12,7	8,2	16,8	−774	1,3	32,9	MSCNS
10	M	11,6	5,7	19,1	0,527	1,4	23,2	*Bacillus sonorensis*
11	M	2	10,8	17,8	1,648	0,01	7,2	MRCNS
12	F	3	6,1	17,3	1,266	1,9	12,2	MSCNS

*F* Female, *M* Male, *MSCNS* Methicillin-susceptible coagulase negative staphylococci*, MRCNS* Methicillin-resistant coagulase negative staphylococci, *MSSA* Methicillin-sensitive *staphylococcus aureus*

The methicillin susceptible coagulase negative staphylococci (MSCNS) colonized 75% of all colonized sensors. Single sensors were colonized by the bacterium of *Klebsiella pneumoniae,* *Ochrobactrum tritici, Bacillus sonorensis* and methicillin-resistant coagulase-negative staphylococci (MRCNS) colonization. It is interesting, that *Ochrobactrum tritici* usually isolated from environmental sources, extremely rare to infect humans colonized electrode in a 9.5-years-old boy training cycling [11].

One electrode was colonized by the mixed flora *Enterococcus faecalis,* MSCNS*, Pseudomonas stutzeri,* MSSA. There were no significant differences between girls and boys in sensor colonization (*p* = 0.524). Patients with sensor colonization did not differ from patients without colonization with HbA1c (6, 85% (6, 3–7, 6%) versus 6, 3% (5, 8–7, 5%): *p* = 0.31). The groups of patients (colonized vs non-colonized) did not differ in insulin intake per day per kg body weight (0.667 j/kg (0.328–0.749 j/kg) versus 0.766 j/kg (0.469–0.911 j/kg); *p* = 0.162). Bacterial colonization of sensors was significantly more frequent in older patients (11.43 years (6.52–12.27 years) versus 8.42 years (3.098–9.375 years); *p* = 0.033). Likelihood of having higher BMI was observed in patients with colonization of sensors but it was not statistically significant 17.10 kg/m^2^ (16.28–18.62 kg/m^2^) versus 15.98 kg/m^2^ (15.14–17.96 kg/m^2^); *p* = 0.109). Sensor colonization was not observed more frequently in patients with longer lasting diabetes (0.88 years (0.027–5.375years) versus 1.611 years (0.945–5.204 years); *p* = 0.341).

The higher frequency of infections in diabetic patients is caused by a hyperglycemic environment, which promotes immune dysfunction. People with diabetes are more prone to infection of the skin and soft tissues, such as folliculitis, furuncles and subcutaneous abscesses [12]. These infections may occur during illness or may be the first symptom of it. Farshchian et al. assessed the occurrence of dermatological disorders in patients with diabetes who attended the Dermatology and Diabetes Clinic of the Medical University in Hamedan. Patients were additionally evaluated for glycemic control and other diabetes-related complications. Skin symptoms were present in 110 of 155 (71%) patients with diabetes. The most common skin lesions in both patients with type 1 and type 2 diabetes were infection related (72%). There were no statistically significant differences in skin symptoms between the two types of diabetes [13]. Simonsen et al. evaluated the occurrence of bacterial infections and their association with chronic hyperglycaemia and diabetic nephropathy in patients with type 1 diabetes. The study was based on Finnish nationwide registers from 1996–2009, they included patients with type 1 diabetes (*n* = 4748) and without diabetes (*n* = 12,954). The authors showed a significantly higher incidence of bacterial infections in patients with type 1 diabetes compared to age-matched sex and non-diabetic patients, which correlated with the severity of diabetic nephropathy [14]. Pam et al. evaluated the bacterial flora of the skin in 50 people with diabetes and 44 without. The authors observed that *Staphylococcus epidermidis* was the predominant strain isolated from the skin of diabetic patients (27 cases). *Staphylococcus aureus* was the second most common in both groups. In the control group, the predominant isolated bacterium was *Escherichia coli* (12 cases). The researchers concluded that there is a change in the bacterial flora of the skin in patients with diabetes mellitus from *Escherichia coli*, which is most common in the control group against *Staphylococcus epidermidis*, which is the most prevalent among patients with diabetes. The authors stated that it may explain the increased incidence of bacterial skin infection in diabetics [15]. Up to now, there have not been many studies assessing bacterial strains colonizing the sensor electrodes of CGM. In our study, the methicillin-sensitive coagulase negative staphylococci (MSCNS) colonized 75% of all colonized sensors.

Jarosz-Chobot et al. investigated the colonization of the catheter in the case of continuous subcutaneous insulin infusion (CSII) in children of the 43 catheters examined, in 7 cases coagulase-negative staphylococci were isolated and in 2 cases mixed flora (both *S. epidermidis* and *S. aureus*) [16]. The authors observed a relationship between colonization of the catheter and elevated HbA1c. Nowakowska et al. examined 141 catheters from 94 children. 34 catheters (24.1%) from 30 children (31.9%) were colonized. In 29 cases catheter tips were colonized by one bacterial species, in 5 cases there was a mixed bacterial flora. Coagulase-negative staphylococci (30), *Staphylococcus aureus* (7), *Corynebacterium jeikeium* (1) and *Kocuria* sp. (2) were isolated. Researchers have shown poorer metabolic control in people with colonization of the catheter (*p* = 0.0355) [17].

In our study, we revealed CGM sensor colonization among older children. The skin flora changes significantly with age, which can naturally affect the habitat for various bacterial species. Puberty and changes in skin physiology are probably important causes of changes in the composition and diversity of the microbial community [18].

Our results do not confirm the relation with higher HbA1c concentrations. The most possible cause is due to the small study group, which is a limitation of our study. The outcomes of our preliminary study suggest that the further expanded microbiological tests on patients' CGM Enlite Medtronic electrodes should be conducted in a representative group of children with diabetes type 1. The further studies should be performed to assess the effect of colonization on the sensor wear duration, calibration problems and whether chronic complications of diabetes affect the colonization of the electrode.

## Conclusions

1. Around 40% of sensors of CGM in diabetic patients are colonized by bacteria.

2. Older children can be more prone to sensor colonization.
